# Supplementary material for: Distinctive roles of translesion polymerases DinB1 and DnaE2 in diversification of the mycobacterial genome through substitution and frameshift mutagenesis
Source: Nat Commun. 2022 Aug 2;13:4493. doi: 10.1038/s41467-022-32022-8 (PMC9346131; doi:10.1038/s41467-022-32022-8)
Supplement: Supplementary file 1 — Supplementary Information [file 41467_2022_32022_MOESM1_ESM.pdf]

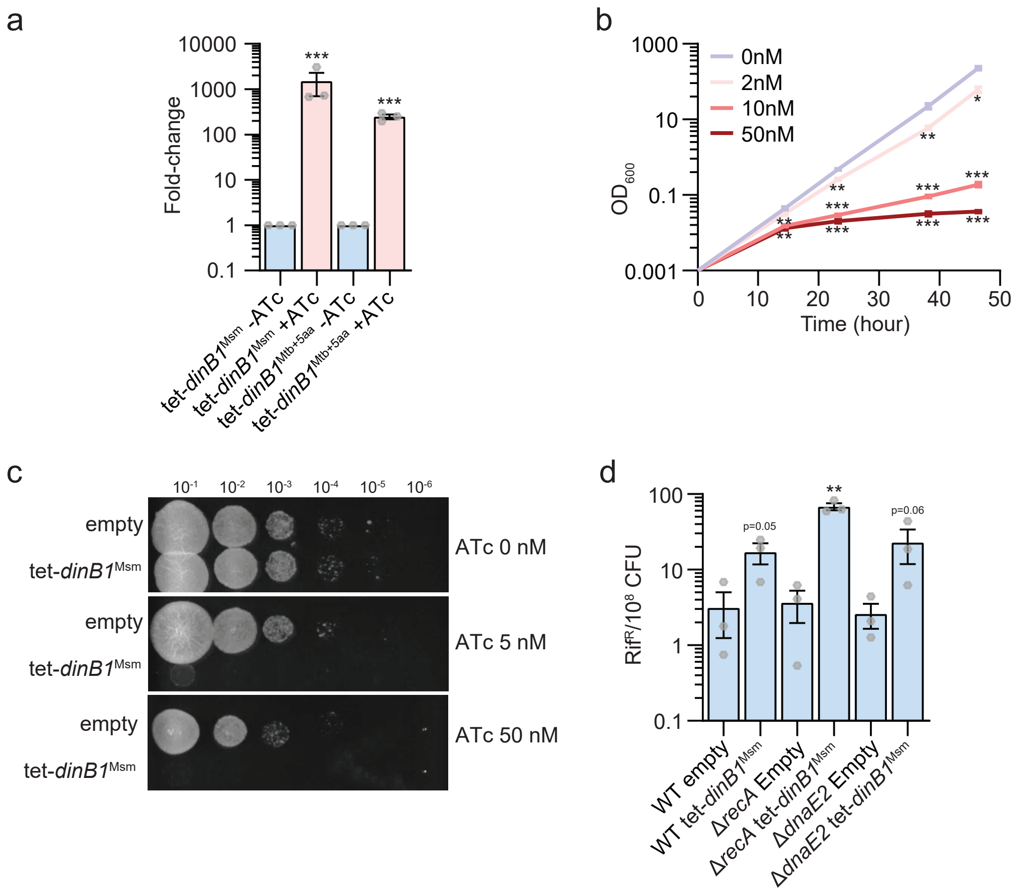

Supplementary Fig. 1 *dinB1* expression phenotypes in WT, Δ*dnaE2* and Δ*recA* backgrounds. a *dinB1*^{Msm} and *dinB1*$^{Mtb+5aa}$ expression measured by RTqPCR in absence (blue) or in presence (red) of inducer. Results are relative expressions compared to untreated condition. **b** Liquid growth of *M. smegmatis* carrying the *dinB1*^{Msm} expression plasmid in presence of the indicated concentrations of inducer. **c** Growth of *M. smegmatis* carrying the *dinB1*^{Msm} expression plasmid on agar medium containing the indicated concentrations of inducer in agar. **d** Rif^{R} frequency in indicated strains in presence of inducer. Results shown are means (± SEM) of data obtained from biological replicates symbolized by grey dots. Stars above the means mark a statistical difference with the reference strain (empty vector or 0nM of inductor) (**, P<0.01; ***, P<0.001). p-values were obtained on log-transformed data by one-way (**a** and **d**) or two-way (**b**) ANOVA with a Bonferroni post-test. Source data, means, SEMs, n numbers, and exact p-values are provided as a Source Data file.

```
Eco  ----------MRKIIHVDMDCFFAAVEMRDNPALRDIPIAIGGSRERRGVISTANYPARK 50
Msm  MEGTVARTASRRWVLHLDMDAFFASVEQLTRPTLRGRPVLVGGLG-GRGVVAGASYEARR 59
Mtb  --------VESRWVLHLDMDAFFASVEQLTRPTLRGRPVLVGGLG-GRGVVAGASYEARA 51
                * ::*:***.***:**   .*:**. *: :**    ***:: *.* **

Eco  FGVRSAMPTGMALKLCPH-LTLLPGRFDAYKEASNHIREIFSRYTSRIEPLSLDEAYLDV 109
Msm  YGARSAMPMHQARRLVGAPAVVLPPRGAVYGVASRRALDTVRSVVPVLEQLSFDEAFGEP 119
Mtb  YGARSAMPMHQARRLIGVTAVVLPPRGVVYGIASRRVFDTVRGLVPVVEQLSFDEAFAEP 111
     :*.*****   * :*     .:** *  .*  **.:  : .   .  :* **:***: :

Eco  TDSVHCH-GSATLIAQEIRQTIFNELQLTASAGVAPVKFLAKIASDMNKPNGQFVITPAE 168
Msm  SELAGAEAADVEAFCERLRAKVLEHTGLVASVGAGSGKQIAKIASGLAKPDGIRVVRREE 179
Mtb  PQLAGAVAEDVETFCERLRRRVRDETGLIASVGAGSGKQIAKIASGLAKPDGIRVVRHAE 171
      : . .   ..  :.:.:*  : :.  * **.*..  * :*****.: **:*  *:   *

Eco  VPAFLQTLPLAKIPGVGKVSAAKLEAMGLRTCGDVQKCDLVMLLKRF-GKFGRILWERSQ 227
Msm  EAVLLHGLPVRKLWGIGPVAEDRLHRIGIETIGAFAALTEAEAANVLGSTIGPALHRLAR 239
Mtb  EQALLSGLPVRRLWGIGPVAEEKLHRLGIETIGQLAALSDAEAANILGATIGPALHRLAR 231
       .:*  **: :: *:* *:  :*. :*:.* * .     .   : : ..:*  * . ::

Eco  GIDERDVNSERLRKSVGVERTMAEDIHHWSECEAIIERLYPELERRLAKVKPDLLIARQG 287
Msm  GIDDRPVAERAEAKQISAESTFPEDLTTLAQLQDAIVPIGEHAHRRLEKDGRGA--RTVT 297
Mtb  GIDDRPVVERAEAKQISAESTFAVDLTTMEQLHEAIDSIAEHAHQRLLRDGRGA--RTIT 289
     ***:* * ..   *.:..* *:  *:    : .  *  :  . .:** :   .

Eco  VKLKFDDFQQTTQE--HVWPRLNKADLIATARKTWDERRGGRGVRLVGLHVTLLDPQMER 345
Msm  VKLKKSDMSTLTRSATLAYATTEASTLIGTARRLLLDPVEIGPIRLVGVGFSGLSDIRQE 357
Mtb  VKLKKSDMSTLTRSATMPYPTTDAGALFTVARRLLPDPLQIGPIRLLGVGFSGLSDIRQE 349
     **** .*:.  *:.    :   : . *: .**:   :      :**:*: .: *.   :.

Eco  QLVLGL------------------------------------------------------ 351
Msm  SLFPDLEQPEEFPDAAPQ-VESVQTGPSSPTTQWRVGDDVAHTELGHGWVQGAGHGVMTV 416
Mtb  SLFADSDLTQETAAAHYVETPGAVVPAAHDATMWRVGDDVAHPELGHGWVQGAGHGVVTV 409
     .*. .
Eco  ----------------------------------------------------------- 351
Msm  RFETRASGPGPARTFPEDSPEISRADPVDSLDWAEYLSSLADYQSTP------------ 463
Mtb  RFETRGSGPGSARTFPVDTGDISNASPLDSLDWPDYIGQLSVEGSAGASAPTVDDVGDR 468
```

DinB1^{Mtb} extra N-ter 5aa

Metal-binding aspartate residues

β-clamp binding domain

	Eco	Msm	Mtb
Eco	100.00	30.17	29.60
Msm	30.17	100.00	74.73
Mtb	29.60	74.73	100.00

Supplementary Fig. 2 DinB1 sequences alignment. CLUSTAL O (1.2.4) multiple sequence alignment of *E. coli* (Eco), *M. smegmatis* (Msm) and *M. tuberculosis* (Mtb) DinB1 sequences.

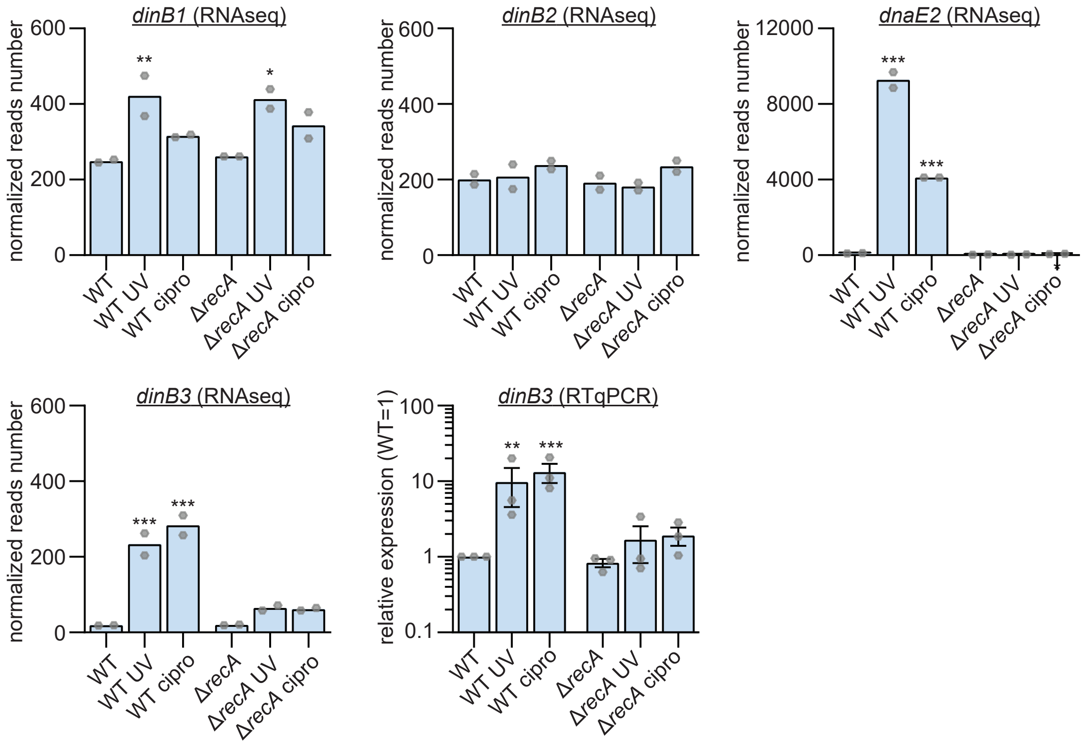

Supplementary Fig. 3 Expression levels of mycobacterial TLS polymerases. Expression level of the indicated *M. smegmatis* genes in indicated genetic backgrounds and conditions measured by RNA sequencing or RT-qPCR. Results shown are means (± SEM) of data obtained from biological replicates symbolized by grey dots. Stars above bars mark a statistical difference with the reference strain (no treatment in the same strain) (*, P<0.05; **, P<0.01; ***, P<0.001). p-values were obtained on log-transformed data by one-way ANOVA with a Bonferroni post-test. Source data, means, SEMs, n numbers, and exact p-values are provided as a Source Data file.

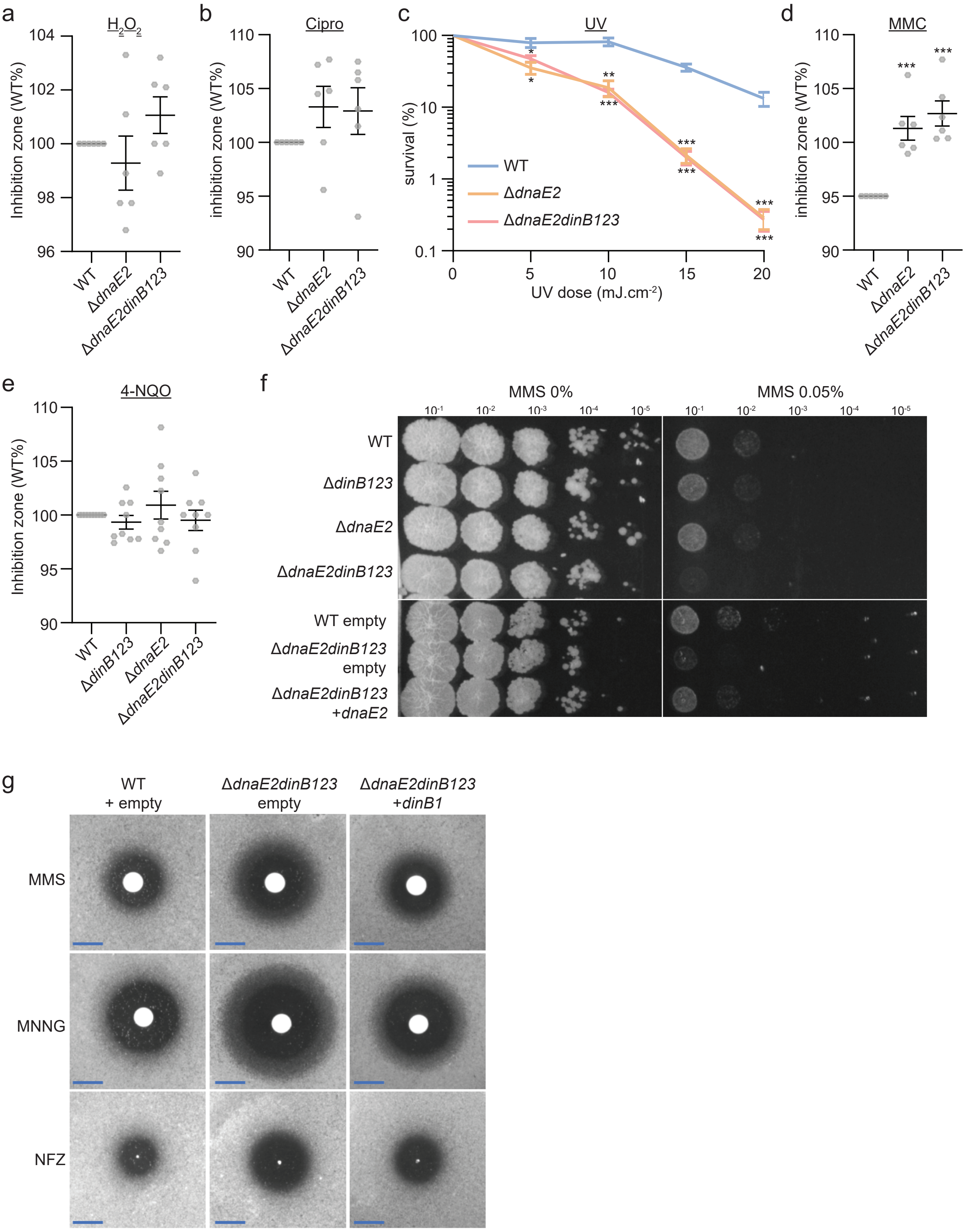

Supplementary Fig. 4 Role of TLS polymerases in DNA damage tolerance. **a** H_2O_2, **b** ciprofloxacin, **d** MMC or **e** 4-NQO sensitivity of indicated strains measured by disc diffusion assay. **c** Viability of indicated strains after treatment with indicated doses of UV. Results shown are means (± SEM) of data obtained from biological replicates symbolized by grey dots. Stars above the means mark a statistical difference with the reference strain (WT) (**, P<0.01; ***, P<0.001). **f** Growth of indicated strains on agar medium containing the indicated concentrations of MMS in agar. **g** Pictures of disc diffusion assays with indicated strains and performed to measure the sensitivity of *M. smegmatis* to the indicated chemical agents. Scale bars indicate 1 cm. p-values were obtained on log-transformed data by one-way (**a**, **b**, **d**, and **e**) or two-way (**c**) ANOVA with a Bonferroni post-test. Source data, means, SEMs, n numbers, and exact p-values are provided as a Source Data file.

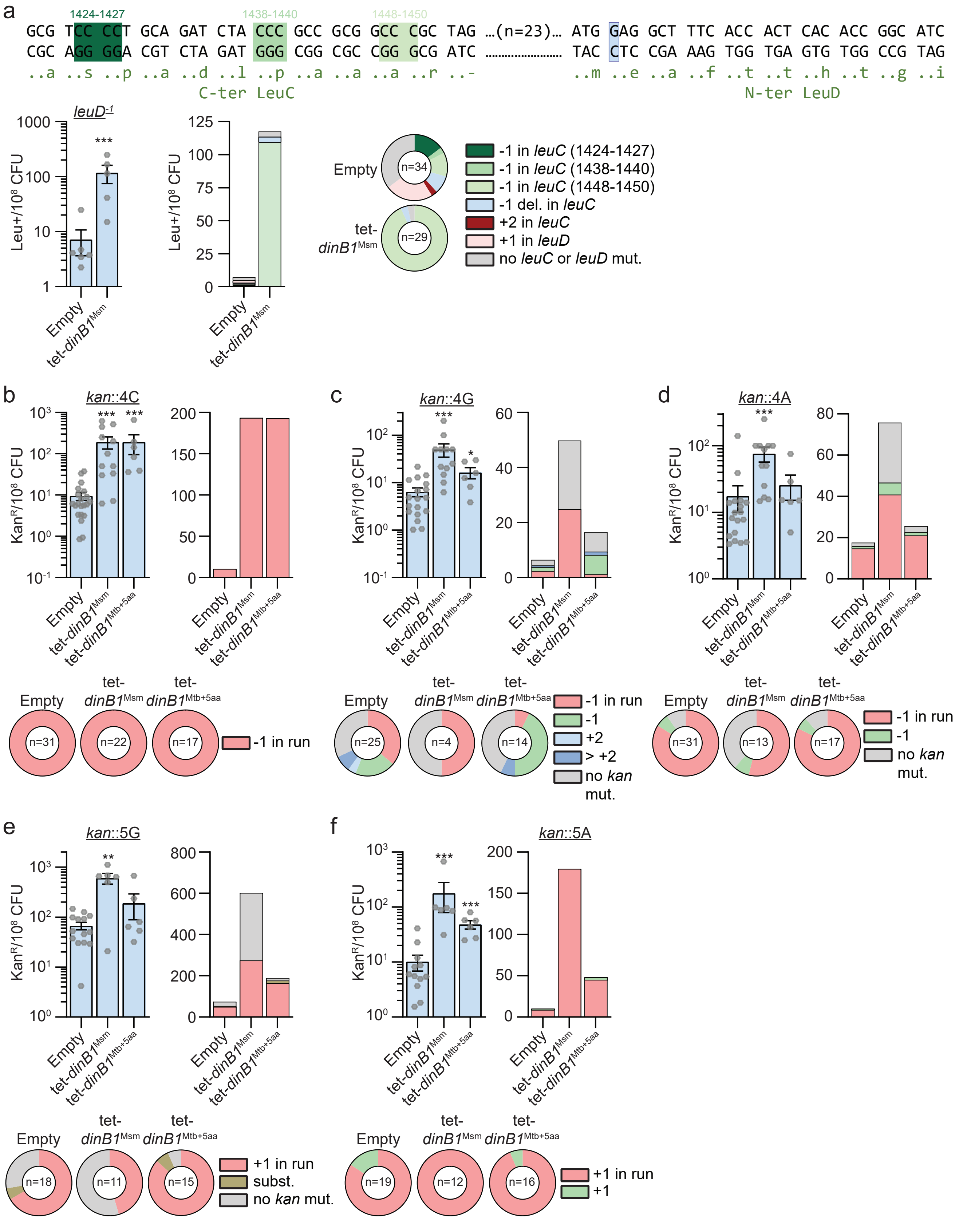

Supplementary Fig. 5 -1 and +1 frameshift mutagenesis in diverse homo-oligonucleotide runs after expression of *dinB1*. a leu+ or (**b**, **c**, **d**, **e** and **f**) kan^R frequency in indicated strains in presence of inducer and relative (pie chart) or absolute (bar chart) frequencies of nucleotide changes detected in *leuD* or *kan* coded by color. The number of sequenced leu$^+$ or kan^R colonies is given in the center of each pie chart. The sequence in **a** shows the *leuD*$^{-1}$ reporter designed to measure +1 frameshift (FS) with the N termini of *leuD* and the C termini of the upstream *leuC*. The blue box shows the 1 base pair deletion introduced to inactivate *leuD* and the green boxes show the homonucleotide runs in which the majority of -1 FS have been detected in leu$^+$ cells. Results shown are means (± SEM) of data obtained from biological replicates symbolized by grey dots. Stars above bars mark a statistical difference with the reference strain (empty) (*, P<0.05; **, P<0.01; ***, P<0.001). p-values were obtained on log-transformed data by one-way ANOVA with a Bonferroni post-test for all graphs except **a** for which a student test was used. Source data, means, SEMs, n numbers, and exact p-values are provided as a Source Data file.

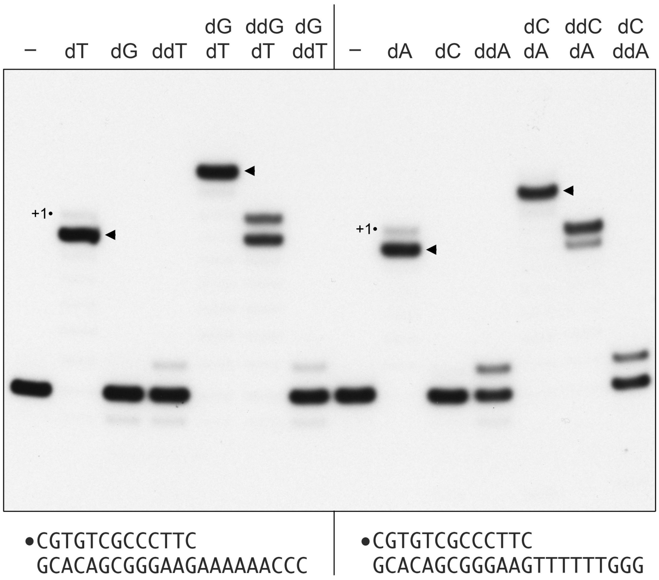

Supplementary Fig. 6 DinB1 discriminates against ddNTPs. Reaction mixtures containing 10 mM Tris-HCl, pH 7.5, 5 mM $MnCl_2$,1 pmol 5' ^{32}P-labeled primer-template DNAs with A6 or T6 runs in the template strand (depicted below, and included as indicated above the lanes), 125 µM nucleotides as specified, and 10 pmol DinB1 were incubated at 37˚C for 15 min. DinB1 was omitted from control reactions in lanes –. The reaction products were analyzed by urea-PAGE and visualized by autoradiography. "Faithful" +6 and +9 primer extension products are denoted by arrowheads.

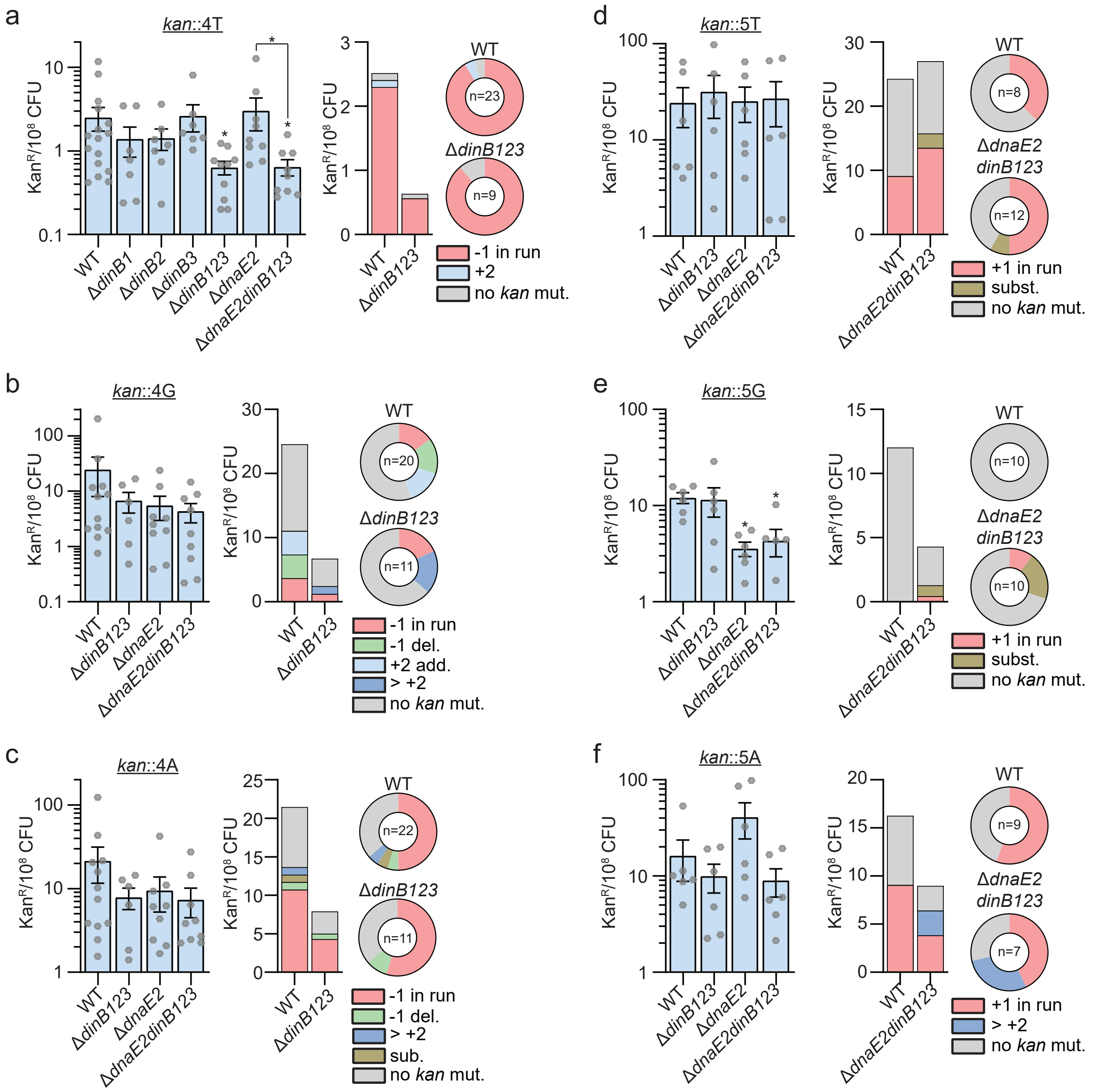

Supplementary Fig. 7 Spontaneous -1 and +1 frameshift mutagenesis in diverse homonucleotide runs. Kan^R frequency in indicated strains and relative (pie chart) or absolute (bar chart) frequencies of nucleotide changes detected in *kan* coded by color. The number of sequenced kan^R colonies is given in the center of each pie chart. Results shown are means (± SEM) of data obtained from biological replicates symbolized by grey dots. Stars above bars mark a statistical difference with the reference strain (WT) (*, $P<0.05$). p-values were obtained on log-transformed data by one-way ANOVA with a Bonferroni post-test. Source data, means, SEMs, n numbers, and exact p-values are provided as a Source Data file.

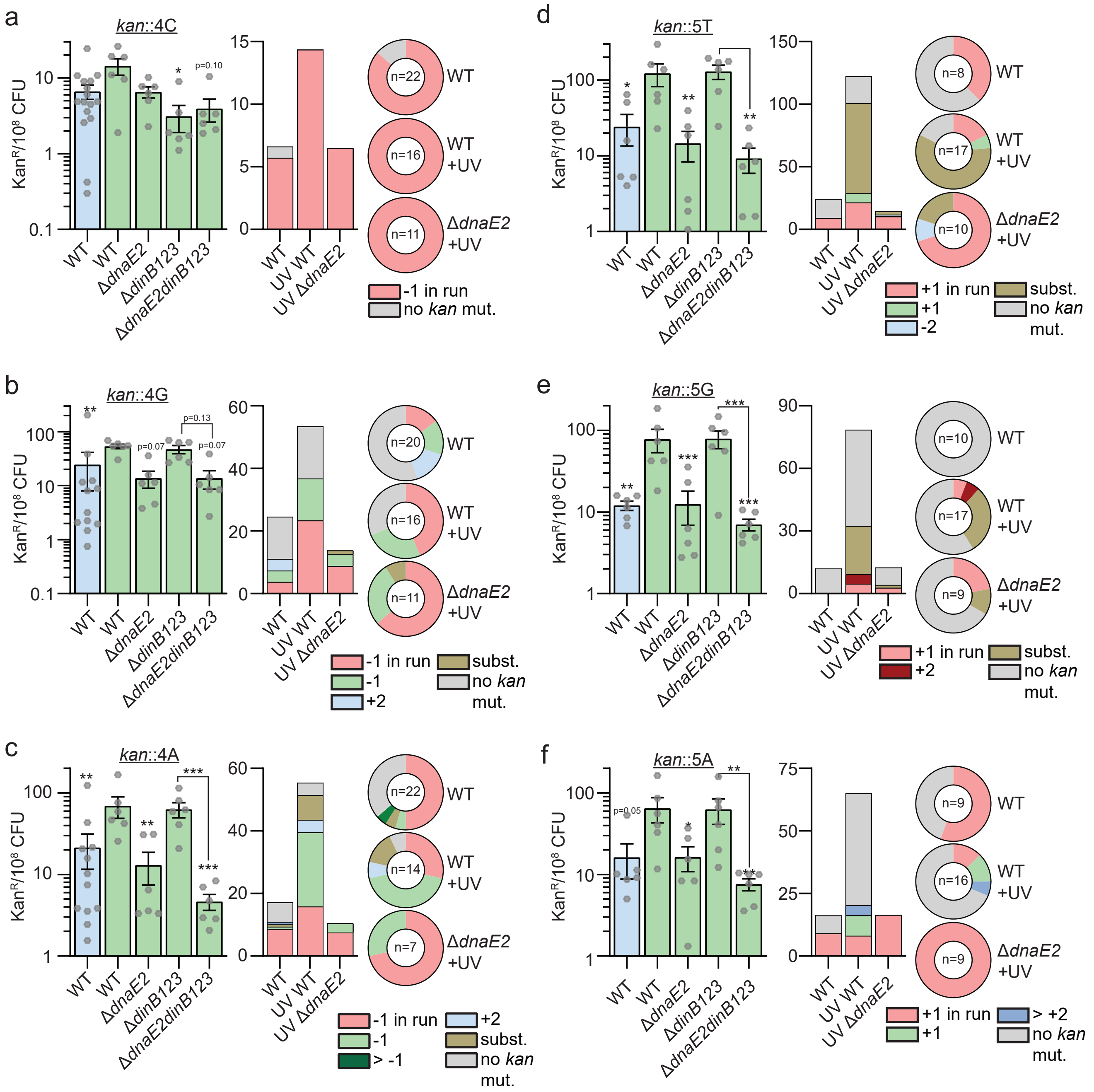

Supplementary Fig. 8 UV-induced -1 and +1 frameshift mutagenesis in diverse homonucleotide runs. Kan^R frequency in indicated strains without (blue) or with UV treatment (green) and relative (pie chart) or absolute (bar chart) frequencies of nucleotide changes detected in *kan* coded by color. The number of sequenced kan^R colonies is given in the center of each pie chart. Results shown are means (± SEM) of data obtained from biological replicates symbolized by grey dots. Stars above bars mark a statistical difference with the reference strain (WT+UV) and lines connecting two strains show a statistical difference between them (*, $P<0.05$; **, $P<0.01$; ***, $P<0.001$). p-values were obtained on log-transformed data by one-way ANOVA with a Bonferroni post-test. Source data, means, SEMs, n numbers, and exact p-values are provided as a Source Data file.

Supplementary tables

Supplementary Table 1: strains used in this work

Strains	Genetic backgrounds	References or sources
M. smegmatis		
PDS1	Wild-type	(Snapper et al., 1990)
PDS122	Δ*dinB2*	(Dupuy et al., 2020)
PDS139	Δ*dnaE2*	(Dupuy et al., 2020)
PDS353	Δ*recA*	(Dupuy et al., 2020)
PDS380	Δ*dinB1*	(Dupuy et al., 2020)
PDS382	Δ*dinB3*	(Dupuy et al., 2020)
PDS388	Δ*dinB1* Δ*dinB2* Δ*dinB3*	(Dupuy et al., 2020)
PDS394	Δ*dnaE2* Δ*dinB1* Δ*dinB2* Δ*dinB3*	(Dupuy et al., 2020)
PDS622	$leuD^{-1}$	This work
PDS630	$leuD^{-2}$	This work
PDS632	$leuD^{-2}$ Δ*dinB2*	This work
PDS684	$leuD^{-2}$ Δ*dnaE2*	This work
PDS686	$leuD^{-2}$ Δ*dinB1*	This work
PDS688	$leuD^{-2}$ Δ*dinB3*	This work
PDS690	$leuD^{-2}$ Δ*dinB123*	This work
PDS692	$leuD^{-2}$ Δ*dnaE2*Δ*dinB2*	This work
E.coli		
DH5α	F^- Φ80*lac*ZΔM15 Δ(*lac*ZYA-*arg*F U169 *rec*A1 *end*A1 *hsd*R17(r_k^-, m_k^+ *pho*A *sup*E44 *thi*-1 *gyr*A96 *rel*A1 λ^-	Lab stock

Supplementary Table 2: plasmids used in this work and cloning methods

Plasmids	Description	Cloning enzyme sites	Cloning primers (infusion reaction)	References or sources
pmsg419	Empty ATc-on system vector (Hyg^R, OriMyc)			Lab Stock
pAJF067	Gene replacement vector (Hyg^R, *galK*, *sacB*)			(Fay and Glickman, 2014)
pDB60	Complementation vector ($Strep^R$, attP(L5))			Lab Stock
pDB60-*dnaE2*	pDB60 derivative for *dnaE2*^{Msm} complementation	*BstB1*	OAM118-OAM185	This work
pDP64	pmsg419 derivative for *dinB1*^{Msm} expression	*Cla1*	ODP290-ODP291	This work
pDP65	pmsg419 derivative for *dinB1*ST expression	*Cla1*	ODP290-ODP292	This work
pDP66	pmsg419 derivative for *dinB1*$^{D113A\text{-}ST}$	*Cla1*	ODP290-ODP293+ODP294-ODP292	This work
pDP88	pmsg419 derivative for *dinB1*^{Mtb} expression	*Cla1*	ODP343-ODP344	This work
pDP104	pAJF067 derivative for *leuD*$^{\Delta 4}$ deletion (*leuD*$^{-1}$)	*Nde1*	ODP389-ODP390+ODP391-ODP392	This work
pDP105	pAJF067 derivative for *leuD*$^{\Delta 4\text{-}5}$ deletion (*leuD*$^{-2}$)	*Nde1*	ODP389-ODP390+ODP393-ODP392	This work
pDP112	pDB60 derivative for *dinB1*^{Msm} complementation	*EcoR1*	ODP480-ODP481	This work
pDP114	pDB60 derivative for *dinB2*^{Msm} complementation	*EcoR1*	ODP425-ODP426+ODP427-ODP428	This work
pDP115	pDB60 derivative for *dinB3*^{Msm} complementation	*EcoR1*	ODP429-ODP430	This work
pDP117	pmsg419 derivative for *dinB1*$^{\Delta\beta clamp}$ expression	*Cla1*	ODP290-ODP433+ODP434-ODP291	This work
pDP118	pDB60 derivative for *dinB1*^{Mtb} complementation	*EcoR1*	ODP435-ODP436	This work
pDP119	pDB60 derivative for *dinB2*^{Mtb} complementation	*EcoR1*	ODP437-ODP438	This work
pDP120	pDB60 derivative with *kan*::3T	*EcoR1*	ODP443-ODP445+ODP446-ODP444	This work
pDP121	pDB60 derivative with *kan*::3C	*EcoR1*	ODP443-ODP445+ODP447-ODP444	This work
pDP122	pDB60 derivative with *kan*::3G	*EcoR1*	ODP443-ODP445+ODP448-ODP444	This work
pDP123	pDB60 derivative with *kan*::3A	*EcoR1*	ODP443-ODP445+ODP449-ODP444	This work
pDP124	pDB60 derivative with *kan*::4T	*EcoR1*	ODP443-ODP445+ODP450-ODP444	This work
pDP125	pDB60 derivative with *kan*::4C	*EcoR1*	ODP443-ODP445+ODP451-ODP444	This work
pDP126	pDB60 derivative with *kan*::4G	*EcoR1*	ODP443-ODP445+ODP452-ODP444	This work
pDP127	pDB60 derivative with *kan*::4A	*EcoR1*	ODP443-ODP445+ODP453-ODP444	This work
pDP128	pDB60 derivative with *kan*::6T	*EcoR1*	ODP443-ODP445+ODP454-ODP444	This work
pDP129	pDB60 derivative with *kan*::6C	*EcoR1*	ODP443-ODP445+ODP455-ODP444	This work
pDP130	pDB60 derivative with *kan*::6G	*EcoR1*	ODP443-ODP445+ODP456-ODP444	This work
pDP131	pDB60 derivative with *kan*::6A	*EcoR1*	ODP443-ODP445+ODP457-ODP444	This work
pDP144	pDB60 derivative with *kan*::5T	*EcoR1*	ODP443-ODP445+ODP490-ODP444	This work
pDP145	pDB60 derivative with *kan*::5C	*EcoR1*	ODP443-ODP445+ODP491-ODP444	This work
pDP146	pDB60 derivative with *kan*::5G	*EcoR1*	ODP443-ODP445+ODP492-ODP444	This work
pDP147	pDB60 derivative with *kan*::5A	*EcoR1*	ODP443-ODP445+ODP493-ODP444	This work
pDP157	pmsg419 derivative for *dinB1*$^{Mtb+5aa}$ expression	*Cla1*	ODP514-ODP344	This work

Supplementary Table 3: primers used in this work

Primers	Sequences (5'→3')*	Targets	Vectors and cloning sites
*dinB*s inducible expression constructs			
ODP290	**CAGAAAGGAGGCCAT**ATGGAGGGCACCGTC	fw *dinB1*^{Msm}	pmsg419 (*ClaI*)
ODP291	**AGGTCGACGGTATCG**CTACGGCGTGCTCTGG	rev *dinB1*^{Msm}	pmsg419 (*ClaI*)
ODP292	**AGGTCGACGGTATCG**CTACTTTTCGAACTGCGGGTGGCTCCACGGCGTGCTCTGGTAG	rev *dinB1*^{Msm} + streptavidin tag	pmsg419 (*ClaI*)
ODP293	GGCGAACGACAGCTGTTCGAGG	rev internal *dinB1*^{Msm} with pol. dead mut. (D113A)	pmsg419 (*ClaI*)
ODP294	**CAGCTGTCGTTCGCC**GAGGCCTTCGGTGAACC	fw internal *dinB1*^{Msm} with pol. dead mut. (D113A)	pmsg419 (*ClaI*)
ODP343	**CAGAAAGGAGGCCAT**GTGCTGCACCTGGACATG	fw *dinB1*^{Mtb}	pmsg419 (*ClaI*)
ODP344	**AGGTCGACGGTATCG**TCACCGGTCGCCGAC	rev *dinB1*^{Mtb}	pmsg419 (*ClaI*)
ODP433	CCGGATATCCGACAGGCCG	internal rev *dinB1*^{Msm} for β clamp binding motif del.	pmsg419 (*ClaI*)
ODP434	CTGTCGGATATCCGGCCGGACCTGGAACAACCCGAG	internal fw *dinB1*^{Msm} for β clamp binding motif del.	pmsg419 (*ClaI*)
ODP514	**CAGAAAGGAGGCCAT**GTGGAGTCCCGCTGGG	fw *dinB1*$^{Mtb+5aa}$	

*15 bp homology with linearized vectors or between two PCR fragments in bold letters.
Streptavidin tag underlined.
Catalytic dead and steric gate mutations in bold letters and underlined.

Primers	Sequences (5'→3')*	Targets	Vectors and cloning sites
leuD$^{\Delta 4}$ or *leuD*$^{\Delta 4\text{-}5}$ constructs			
ODP389	**CTAGTATGCATCATA**AGGCTTTGGCCTACATGGAC	fw *leuD*	pAJF067 (*NdeI*)
ODP390	CATCACGCTTCTCCTTCGTG	internal rev *leuD*	pAJF067 (*NdeI*)
ODP391	AGGAGAAGCGTGATGAGGCTTTCACCACTCACACC	internal fw *leuD* with Δ4 mutation	pAJF067 (*NdeI*)
ODP392	**CTAGGCAATTGCATA**TGTAGTCATCAATCCTGAACGG	rev *leuD*	pAJF067 (*NdeI*)
ODP393	AGGAGAAGCGTGATGGGCTTTCACCACTCACACC	internal fw *leuD* with Δ4-5 mutation	pAJF067 (*NdeI*)

*15 bp homology with linearized vectors in bold letters.
15 bp homology between two PCR fragments underlined

Primers	Sequences (5'→3')*	Targets	Vectors and cloning sites
Δ*dinB*s and Δ*dnaE2* complementation constructs			
OAM118	**GTACCAGATCTTTAA**ATACGATCTGGCGTGCG	fw *dnaE2*^{Msm}	pDB60 (*BstB1*)
OAM185	**CATCGATAAGCTTCA**CTTCTCGAACTGGGGGTGGCTCCACCGGAAGTCGCGGGAG	rev *dnaE2*^{Msm}	pDB60 (*BstB1*)
ODP425	**TCCAGCTGCAGAATT**ATGGTGGCGTTGTACTCGG	fw *dinB2*^{Msm} promoter	pDB60 (*EcoR1*)
ODP426	**CTTATCTGTGAATTT**CTGTGAATCC	rev *dinB2*^{Msm} promoter	pDB60 (*EcoR1*)
ODP427	**AAATTCACAGATAAG**ATGACCAAATGGGTGCTCCAC	fw *dinB2*^{Msm}	pDB60 (*EcoR1*)
ODP428	**GATAAGCTTCGAATT**TCGAGGTTAGGTGCCTGCAG	rev *dinB2*^{Msm}	pDB60 (*EcoR1*)
ODP429	**TCCAGCTGCAGAATT**TGCTGTTGTCGCTGATCGATC	fw *dinB3*^{Msm}	pDB60 (*EcoR1*)
ODP430	**GATAAGCTTCGAATT**TCTTCTAGTCCGGCAGCATGG	rev *dinB3*^{Msm}	pDB60 (*EcoR1*)
ODP435	**TCCAGCTGCAGAATT**ATGGCACCGTCACTGCCGAAC	fw *dinB1*^{Mtb}	pDB60 (*EcoR1*)
ODP436	**GATAAGCTTCGAATT**TCACCGGTCGCCGACGTC	rev *dinB1*^{Mtb}	pDB60 (*EcoR1*)
ODP437	**TCCAGCTGCAGAATT**ACGCAATCGTGCACTCCTGTTG	fw *dinB2*^{Mtb}	pDB60 (*EcoR1*)
ODP438	**GATAAGCTTCGAATT**CTAGGCCAGTTCTAACCGCACTC	rev *dinB2*^{Mtb}	pDB60 (*EcoR1*)
ODP480	**TCCAGCTGCAGAATT**TGAGTTCGACCTCACCGTTGAC	fw *dinB1*^{Msm}	pDB60 (*EcoR1*)
ODP481	**GATAAGCTTCGAATT**TCGACGTGCTGCCGGAAG	rev *dinB1*^{Msm}	pDB60 (*EcoR1*)

15 bp homology with linearized vectors or between two PCR fragments in bold letters.

Primers	Sequences (5'→3')*	Targets	Vectors and cloning sites
kan inactivated by homo-oligonucleotide runs			
ODP443	**TCCAGCTGCAGAATT**TCCCAAGGACACTGAGTCC	fw *kan*	pDB60 (*EcoR1*)
ODP444	**GATAAGCTTCGAATT**TTGCTGACTCATACCAGGC	rev *kan*	pDB60 (*EcoR1*)
ODP445	**CATAACACCCCTTGT**ATTACTG	internal rev *kan*	pDB60 (*EcoR1*)
ODP446	**ACAAGGGGTGTTATG**TTTAGCCATATTCAACGGGAAACG	internal fw *kan* (3T addition)	pDB60 (*EcoR1*)

ODP447	**ACAAGGGGTGTTATG**CCCAGCCATATTCAACGGGAAACG	internal fw *kan* (3C addition)	pDB60 (*EcoR1*)
ODP448	**ACAAGGGGTGTTATG**GGGAAGCCATATTCAACGGGAAACG	internal fw *kan* (3G addition)	pDB60 (*EcoR1*)
ODP449	**ACAAGGGGTGTTATG**GAAAGCCATATTCAACGGGAAACG	internal fw *kan* (3A addition)	pDB60 (*EcoR1*)
ODP450	**ACAAGGGGTGTTATG**TTTTAGCCATATTCAACGGGAAACG	internal fw *kan* (4T addition)	pDB60 (*EcoR1*)
ODP451	**ACAAGGGGTGTTATG**CCCCAGCCATATTCAACGGGAAACG	internal fw *kan* (4C addition)	pDB60 (*EcoR1*)
ODP452	**ACAAGGGGTGTTATG**GGGAAGCCATATTCAACGGGAAACG	internal fw *kan* (4G addition)	pDB60 (*EcoR1*)
ODP453	**ACAAGGGGTGTTATG**GAAAAGCCATATTCAACGGGAAACG	internal fw *kan* (4A addition)	pDB60 (*EcoR1*)
ODP454	**ACAAGGGGTGTTATG**TTTTTTAGCCATATTCAACGGGAAACG	internal fw *kan* (6T addition)	pDB60 (*EcoR1*)
ODP455	**ACAAGGGGTGTTATG**CCCCCCAGCCATATTCAACGGGAAACG	internal fw *kan* (6C addition)	pDB60 (*EcoR1*)
ODP456	**ACAAGGGGTGTTATG**GGGGGAAGCCATATTCAACGGGAAACG	internal fw *kan* (6G addition)	pDB60 (*EcoR1*)
ODP457	**ACAAGGGGTGTTATG**GAAAAAAGCCATATTCAACGGGAAACG	internal fw *kan* (6A addition)	pDB60 (*EcoR1*)
ODP490	**ACAAGGGGTGTTATG**TTTTTAGCCATATTCAACGGGAAACG	internal fw *kan* (5T addition)	pDB60 (*EcoR1*)
ODP491	**ACAAGGGGTGTTATG**CCCCCAGCCATATTCAACGGGAAACG	internal fw *kan* (5C addition)	pDB60 (*EcoR1*)
ODP492	**ACAAGGGGTGTTATG**GGGGAAGCCATATTCAACGGGAAACG	internal fw *kan* (5G addition)	pDB60 (*EcoR1*)
ODP493	**ACAAGGGGTGTTATG**GAAAAAGCCATATTCAACGGGAAACG	internal fw *kan* (5A addition)	pDB60 (*EcoR1*)

*15 bp homology with linearized vectors or between two PCR fragments in bold letters.
homo-oligonucleotide runs underlined.

Screening PCR and sequencing

ODP236	CTCCCTATCAGTGATAGATAGGCTCTGG	fw PCR screening and seq pmsg419 cloning
ODP237	CATGACCAACTTCGATAACGTTCTCGG	rev PCR screening and seq pmsg419 cloning
ODP474	TGATTCTGTGGATAACCGTATTACCGCCTTTG	fw PCR screening and seq pDB60 cloning
ODP475	AAGGCCCAGTCTTTCGACTGAGC	rev PCR screening and seq pDB60 cloning
ODP169	ATCTCTCCGGCTTCACCG	fw PCR screening and seq pAJF067 cloning
ODP170	AACGCCAGCAACGCGG	rev PCR screening and seq pAJF067 cloning
ODP378	CAAGAAGCTGGGCCTGAACGC	fw *rpoB* PCR
ODP379	GCGGTTGGCGTCGTCGTG	rev *rpoB* PCR
ODP380	GAGCGTGTCGTGCGTGAG	*rpoB* seq
ODP395	TTCAGGCGAAGCTAGCGAAC	rev *leuD* PCR
ODP398	ACCACGTTCGAGTTCCTCAAGG	fw *leuC* PCR
ODP399	TCCAACCGCAACTTCGAGG	*leuC-leuD* seq
ODP476	TGGCCTTTTGCTGGCCTTTTGC	fw *kan* PCR
ODP477	TTCAACAAAGCCGCCGTCCC	rev *kan* PCR
ODP479	ACTGAATCCGGTGAGAATGG	*kan* seq

In vitro DNA slippage assay

SG-FS1	CGTGTCGCCCTTC	5' ^{32}P-labeled primer DNA strand
SG-FS1	GGGTTTTGAAGGGCGACACG	unlabeled template strand (4T)
SG-FS1	GGGTTTTTTGAAGGGCGACACG	unlabeled template strand (6T)
SG-FS1	GGGTTTTTTTTGAAGGGCGACACG	unlabeled template strand (8T)
SG-FS1	CCCAAAAGAAGGGCGACAC	unlabeled template strand (4A)
SG-FS1	CCCAAAAAAGAAGGGCGACAC	unlabeled template strand (6A)
SG-FS1	CCCAAAAAAAAGAAGGGCGACAC	unlabeled template strand (8A)

*homo-oligonucleotide runs underlined.

Supplementary Table 4: *rpoB* mutations incorporated by TLS polymerases

	Empty		*dinB1*[Msm] OE		*dinB1*[Mtb+5aa] OE		Δ*dnaE2* H_2O_2		WT H_2O_2	
mutations	prop. in %	mut./10^8	prop. in %	mut./10^8	prop. in %	mut./10^8	prop. in %	mut./10^8	prop. in %	mut./10^8
Leu427(CTG>CCT)	0.00	0.00	0.00	0.00	0.00	0.00	6.82	1.30	4.00	3.00
Ser428(TCG>TGG)	0.00	0.00	0.00	0.00	0.00	0.00	9.09	1.73	2.00	1.50
Gln429(CAG>AAG)	2.17	0.12	0.00	0.00	4.17	1.84	2.27	0.43	2.00	1.50
Gln429(CAG>CTG)	0.00	0.00	2.22	0.76	0.00	0.00	0.00	0.00	4.00	3.00
Gln429(CAG>CCG)	1.09	0.06	0.00	0.00	0.00	0.00	0.00	0.00	0.00	0.00
Asp432(GAC>TAC)	2.17	0.12	0.00	0.00	0.00	0.00	2.27	0.43	0.00	0.00
Asp432(GAC>AAC)	0.00	0.00	0.00	0.00	0.00	0.00	2.27	0.43	0.00	0.00
Asp432(GAC>GTC)	2.17	0.12	0.00	0.00	0.00	0.00	0.00	0.00	2.00	1.50
Asp432(GAC>GGC)	2.17	0.12	4.44	1.53	0.00	0.00	0.00	0.00	0.00	0.00
Asp432(GAC>GAG)	0.00	0.00	0.00	0.00	0.00	0.00	0.00	0.00	0.00	0.00
Asp432(GAC>TTC)	0.00	0.00	0.00	0.00	0.00	0.00	0.00	0.00	2.00	1.50
Asn435(AAC>AAG)	0.00	0.00	0.00	0.00	0.00	0.00	0.00	0.00	6.00	4.49
Ser438(TCG>TTG)	16.30	0.90	2.22	0.76	4.17	1.84	2.27	0.43	12.00	8.99
Ser438(TCG>TGG)	0.00	0.00	0.00	0.00	0.00	0.00	0.00	0.00	4.00	3.00
His442(CAC>TAC)	15.22	0.84	6.67	2.29	0.00	0.00	9.09	1.73	10.00	7.49
His442(CAC>AAC)	0.00	0.00	0.00	0.00	0.00	0.00	0.00	0.00	2.00	1.50
His442(CAC>GAC)	9.78	0.54	0.00	0.00	4.17	1.84	9.09	1.73	8.00	5.99
His442(CAC>CCC)	3.26	0.18	4.44	1.53	0.00	0.00	0.00	0.00	2.00	1.50
His442(CAC>CGC)	21.74	1.19	73.33	25.21	75.00	33.19	2.27	0.43	2.00	1.50
His442(CAC>CCG)	0.00	0.00	0.00	0.00	0.00	0.00	0.00	0.00	2.00	1.50
Arg445(CGT>TGT)	0.00	0.00	0.00	0.00	0.00	0.00	2.27	0.43	4.00	3.00
Arg445(CGT>CTT)	0.00	0.00	2.22	0.76	0.00	0.00	0.00	0.00	0.00	0.00
Arg445(CGT>CCT)	0.00	0.00	0.00	0.00	0.00	0.00	2.27	0.43	2.00	1.50
Ser447(TCG>TTG)	4.35	0.24	0.00	0.00	0.00	0.00	22.73	4.33	16.00	11.98
Ser447(TCG>TGG)	0.00	0.00	0.00	0.00	4.17	1.84	13.64	2.60	2.00	1.50
Leu449(CTG>CCG)	4.35	0.24	2.22	0.76	0.00	0.00	2.27	0.43	0.00	0.00
Gly450(GGC>AGC)	0.00	0.00	0.00	0.00	0.00	0.00	0.00	0.00	2.00	1.50
Gly450(GGC>TGC)	1.09	0.06	0.00	0.00	0.00	0.00	0.00	0.00	0.00	0.00
Pro480(CCT>CTT)	1.09	0.06	0.00	0.00	0.00	0.00	0.00	0.00	0.00	0.00
Ile488(ATC>TTC)	1.09	0.06	0.00	0.00	4.17	1.84	0.00	0.00	0.00	0.00
Ile488(ATC>ATG)	0.00	0.00	0.00	0.00	0.00	0.00	2.27	0.43	0.00	0.00
Ser490(TCG>TTG)	0.00	0.00	0.00	0.00	0.00	0.00	2.27	0.43	2.00	1.50
del.	1.09	0.06	0.00	0.00	0.00	0.00	0.00	0.00	0.00	0.00
no *rpoB* mut.	10.87	0.60	2.22	0.76	4.17	1.84	6.82	1.30	8.00	5.99
total	100.00	5.49	100.00	34.38	100.00	44.26	100.00	19.07	100.00	74.90

prop. in %: relative frequency of *rpoB* mutations found in indicated strains (also shown in Figures 2C and 3C).
mut./10^8: absolute frequency of *rpoB* mutations, expressed in number of mutations per 10^8 CFU found in indicated strains (also shown in Figures 2D and 3C).

Supplementary Table 5: *leuD* mutations detected in indicated strains

leuD^{-2} mut.	empty	tet-*dinB1*^{Msm}	tet-*dinB1*$^{Mtb+5aa}$	WT	Δ*dinB1*	Δ*dinB2*	Δ*dinB3*	Δ*dinB123*	Δ*dnaE2*	Δ*dnaE2*Δ*dinB123*	WT+empty	Δ*dinB1*+empty	Δ*dinB1*+*dinB1*	WT UV	Δ*dnaE2* UV	Δ*dinB123* UV	Δ*dinB123*Δ*dnaE2* UV
T del. (nuc. 9-11)	18	20	11	33		23	22	3	14	7	10	2	19	5	6	5	0
A del. (nuc. 1)								1									
T del. (nuc. 2)						1						4					
G del. (nuc. 3)				2	3	2		1	1			1	1			1	
C del. (nuc. 8)				1				2	1							1	
C del. (nuc. 12)		2		4				2									
A del. (nuc. 13)	1																
C del. (nuc. 14)					1	1	1	1				1					
A del. (nuc. 16)							1							1			
C del. (nuc. 17)													3				
T del. (nuc. 18)		1		2				1									
C del. (nuc. 19)												1		1			
A del. (nuc. 20)				7	3	1	1	3		2	1		2		1		
C del. (nuc. 21)						1		2			1		1				
A del. (nuc. 22)							4	2	1								
C del. (nuc. 23)				2		2	1								1		
> -1 del	2			1	1			1		2				1	0	0	0
+2 add.	4			3	3		3	3	1	4	3	0		0	3	2	2
> +2 add.	3			4	3	1	1	2	1	0	2	3		1	0	3	1
subst.				5			3	13	3	3	5	0	3	5	4	6	4
no *leuD* mut.	13	3	1	18	14	0	5	12	18	16	11	17	6	10	9	6	11
Total	41	26	12	82	28	32	42	49	40	34	33	29	35	24	24	24	18

Supplementary Table 6: DinB1 and DnaE2 mutation spectrum compared to *rpoB* mutations found in Mtb clinical isolates.

M. smegmatis					*E. coli*	Mtb		WHO report (rif^R clin. isol.)		
amino acids	mut. (aa)	mut. (codon)	freq. empty	freq. *dinB1*$^{Mtb+5aa}$ OE	aa	aa	codon	mut.	freq.	total nbr.
His442(H)	His(H)>Arg(R)	CAC>CGC	1.19	33.19	His526	His445	CAC	H445R	0.8%	79

M. smegmatis					*E. coli*	Mtb		WHO report (rif^R clin. isol.)		
amino acids	mut. (aa)	mut. (codon)	freq. Δ*dnaE2* H_2O_2	freq. WT H_2O_2	aa	aa	codon	mut.	freq.	total nbr.
Ser447(S)	Ser(S)>Leu(L)	TCG>TTG	4.33	11.98	Ser531	Ser450	TCG	S450L	66.2%	6536
Ser438(S)	Ser(S)>Leu(L)	TCG>TTG	0.43	8.99	Ser522	Ser441	TCG	S441L	0.3%	26
his442(H)	His(H)>Tyr(Y)	CAC>TAC	1.73	7.49	His526	His445	CAC	H445Y	3.5%	347
his442(H)	His(H)>Asp(D)	CAC>GAC	1.73	5.99	His526	His445	CAC	H445D	2.9%	288
Asn435(N)	Asn(N)>Lys(K)	AAC>AAG	0.00	4.49	Asn519	Asn438	AAC	N438K	ND	ND
Leu427(L)	Leu(L)>Pro(P)	CTG>CCT	1.30	3.00	Leu511	Leu430	CTG	L430P	1.1%	106
Gln429(Q)	Gln(Q)>Leu(L)	CAG>CTG	0.00	3.00	Gln513	Gln432	CAA	Q432L	ND	ND
Ser438(S)	Ser(S)>Trp(W)	TCG>TGG	0.00	3.00	Ser522	Ser441	TCG	S441W	ND	ND
Arg445(S)	Arg(R)>Cys(C)	CGT>TGT	0.43	3.00	Arg529	Arg448	CGA	R448C	ND	ND

Freq.: absolute frequency of *rpoB* mutations, expressed in number of mutation per 10^8 CFU found in indicated strains (also shown in Figures 2D and 3C).
a.a.: *rpoB* amino acids of *M. smegmatis* found in rif^R colonies and corresponding amino acids in the *E. coli* and Mtb *rpoB* gene.
WHO report: frequencies (freq.) and number (total nbr.) of Mtb rif^R clinical isolates in which listed *rpoB* mutations have been detected among around 10 000 sequenced Mtb rif^R clinical isolates. The data are published in the WHO mutations catalogue, 2021.
The mutations listed in this table are the mutations detected at a frequency $\geq 3/10^8$ CFU in *M. smegmatis* the condition in which the TLS polymerase is expressed.

References of supplementary tables:

Dupuy, P., Howlader, M., and Glickman, M.S. (2020). A multilayered repair system protects the mycobacterial chromosome from endogenous and antibiotic-induced oxidative damage. PNAS *117*, 19517–19527.

Fay, A., and Glickman, M.S. (2014). An essential nonredundant role for mycobacterial DnaK in native protein folding. PLoS Genet. *10*, e1004516.

Snapper, S.B., Melton, R.E., Mustafa, S., Kieser, T., and Jacobs, W.R. (1990). Isolation and characterization of efficient plasmid transformation mutants of Mycobacterium smegmatis. Mol. Microbiol. *4*, 1911–1919.
